# Changing User Experience of Wearable Activity Monitors Over 7 Years: Repeat Cross-Sectional Survey Study

**DOI:** 10.2196/56251

**Published:** 2025-02-13

**Authors:** Darcy Beckett, Rachel Curtis, Kimberley Szeto, Carol Maher

**Affiliations:** 1 Alliance for Research in Exercise, Nutrition and Activity Allied Health and Human Performance University of South Australia Adelaide Australia

**Keywords:** cross-sectional survey, activity tracker, user experience, physical activity, sleep, diet, health behaviour, wearable activity trackers, cohort, Apple, Fitbit, preferences, reliability, accessibility, lifestyle, mobile phone

## Abstract

**Background:**

Lifestyle behaviors, including physical inactivity, sedentary behavior, poor sleep, and unhealthy diet, significantly impact global population health. Wearable activity trackers (WATs) have emerged as tools to enhance health behaviors; however, their effectiveness and continued use depend on their user experience.

**Objective:**

This study aims to explore changes in user experiences, preferences, and perceived impacts of WATs from 2016 to 2023.

**Methods:**

We conducted a cross-sectional online survey among an international cohort of adults (n=475, comprising 387 current and 88 former WAT users). Results were compared with a 2016 cross-sectional online survey (n=237, comprising 200 current and 37 former WAT users) using descriptive statistics and chi-square tests. The survey examined brand preference, feature usefulness, motivations, perceived health behavior change, social sharing behaviors, and technical issues.

**Results:**

In 2023, Apple (210/475, 44%) and Fitbit (101/475, 21%) were the most commonly used devices, compared with the 2016 survey where Fitbit (160/237, 68%) and Garmin devices (39/237, 17%) were most common. The median usage duration in 2023 was 18 months, significantly longer than the 7 months reported in 2016, with most users planning ongoing use. Users in both survey years reported greater improvements in physical activity than diet or sleep, despite lower improvement in physical activity in 2023 compared with 2016, contrasted with greater perceived improvements in diet and sleep. Social media sharing of WAT data notably rose to 73% (283/387) in 2023 from 35% (70/200) in 2016. However, reports of technical issues and discomfort increased, alongside a decrease in overall positive experiences. There was also a noticeable shift in discontinuation reasons, from having learned everything possible in 2016 to dissatisfaction in 2023.

**Conclusions:**

The study highlights significant shifts in WAT usage, including extended use and evolving preferences for brands and features. The rise in social media sharing indicates a deeper integration of WATs into everyday life. However, user feedback points to a need for enhanced design and functionality despite technological progress. These findings illustrate WAT’s potential in health promotion, emphasizing the need for user-focused design in diverse populations to fully realize their benefits in enhancing health behaviors.

## Introduction

Lifestyle behaviors such as physical inactivity, sedentary behavior, poor sleep quality, and unhealthy dietary habits collectively contribute significantly to global morbidity and mortality [1-4]. Current estimates suggest that 1.4 billion adults, accounting for 27.5% of the global adult population, do not adhere to recommended physical activity guidelines [1], increasing the risk of major noncommunicable diseases, including coronary heart disease, stroke, type 2 diabetes, cancers, and mental illness [5]. Sedentary behavior, which is considered a distinct health risk factor, also contributes to adverse health outcomes [6]. In addition, inadequate sleep [7] and suboptimal dietary patterns [8] are recognized as critical determinants of poor health, influencing both physical and mental well-being. Together, these lifestyle factors form a quartet of behaviors that significantly impact global health, underscoring the need for effective interventions and public health strategies. Observation of daily living research emphasizes the importance of individual’s understanding of their everyday activities and their impact on their health [9]. Wearable activity trackers (WATs) are one method of tracking activity, providing health insights and highlighting the potential for targeted interventions to improve health and well-being.

WATs have emerged as promising tools for enhancing health behaviors. The WAT market is experiencing rapid growth, valued at US $53.94 billion in 2023, and is projected to grow from US $62.03 billion in 2024 to US $290.85 billion by 2032 [10]. These devices, often worn on the wrist, are designed to positively influence daily lifestyle behaviors by providing users with a variety of health-related data, including metrics such as step count, calories burned, heart rate, sleep patterns, stairs climbed, and sedentary time. Assessment of physical activity using WATs tends to perform better in terms of validity and reliability than self-report assessment [11,12], pointing to their potential to improve activity surveillance and understanding of how activity can impact health. Beyond tracking data, WATs also incorporate behavior change techniques like goal setting, feedback on progress, action planning, review of behavior goals, the discrepancy between current behavior and goal, self-monitoring of behavior, biofeedback, commitment, social support, and rewards for achieving daily objectives, all designed to promote healthier behaviors [13].

The effectiveness of WATs in increasing physical activity levels and improving health across all age groups and in both healthy and clinical populations is well-documented. An umbrella review encompassing 719 primary studies demonstrated that WAT-based interventions significantly improved physical activity, daily step count, time spent in moderate to vigorous physical activity, body composition, blood pressure, and fitness among 163,992 participants [14]. There is also emerging evidence regarding WAT’s role in sleep improvement, with a meta-analysis of 20 randomized controlled trials suggesting significant improvements in sleep quality parameters [15]. Furthermore, extensive research has been conducted on the reliability and validity of WATs [16,17].

However, there has been relatively less focus on users’ perceptions and experiences of WATs. A 2016 study with 133 participants recruited through Amazon Mechanical Turk explored the well-being implications of these devices, revealing that WATs could impact users’ daily life experiences beyond behavior change, providing psychological benefits and fostering a sense of relatedness [18]. Our group conducted a cross-sectional survey in 2016 with 237 Australian adults, examining user experiences with WATs, including usage patterns, social media data sharing, perceived behavior changes, and technical challenges [19]. We also investigated the emotional and psychological responses of users to their activity trackers, finding that most users felt empowered and motivated, while some experienced negative emotions such as anxiety or frustration [20]. Systematic literature reviews of studies conducted up until 2017 [21], 2019 [22], and 2020 [23] have examined various themes related to acceptability and user experience using quantitative and qualitative methods. They identified key issues in WAT user experience, including preferred WAT features [23], how user motivation impacts user experience [23], and how acceptance and usability research provide important design implications [21]. Since these studies, WATs have become more prevalent, and more diverse populations may now be using them. With the regular release of new models featuring advanced capabilities, user experience with WATs is likely to have evolved.

This study aims to understand current and former users’ experiences of WATs in 2023 and compare these with our 2016 survey findings. Specifically, we will explore (1) patterns of use (eg, length of use, features used), (2) perceived motivation and behavior change, (3) practices and reasons underpinning data sharing through social media, and (4) technical issues and other barriers to use. By comparing these results with our previous survey [19], we hope to provide insights that will assist clinicians in supporting patients through observation of daily living, as well as aid researchers in exercise physiology, physical activity, and digital health considering WAT use in clinical or general populations.

## Methods

### Overview

### Survey Instrument Development

An online survey instrument (Multimedia Appendix 1) was developed to address the research objectives and was delivered through Qualtrics. To allow for comparison, the survey items were based on the Maher et al [19] 2016 survey, which was developed in consultation with experts and showed test-retest bivariate correlation coefficients ranging between *r*=0.30 and 0.81 for survey sections [19]. A total of 7 items were added or edited to reflect recent advances in WAT features, such as meditation, workouts, phone call or text messaging, contactless payments, and calendar reminders. WATs were defined in the survey as any bodily-worn device that objectively measures physical activity and provides feedback [24]. The test-retest reliability of the 2023 survey was evaluated with a sample of 19 individuals (14 females, 4 males, and 1 nonbinary or third gender) recruited through Facebook over a 7 to 21-day interval, yielding bivariate correlation coefficients between *r*=0.45 and 0.79 across sections.

### Participants

#### Overview

Participants were adults aged 18 and over who were either current or former WAT users within the past 3 years and had used the device for at least 1 month. Exclusion criteria included the use of health-related smartphone applications or software without an associated WAT, WATs not measuring or providing physical activity feedback, and WATs not interacting with a smartphone or computer.

To recruit a diverse, international participant pool, voluntary response sampling was used using 2 platforms:

1. Amazon Mechanical Turk (MTurk): An Amazon-hosted crowdsourcing service that offers rapid access to a varied participant base. For this study, the Qualtrics survey link was posted on MTurk.
2. Facebook: The survey was disseminated through various community groups, the university research center’s social media pages, and the research team’s profiles. To encourage broader sharing, Facebook users who shared the post were entered into a draw to win one of 2 Aus $100 (US $62.83) retail gift vouchers.

#### Demographic Characteristics and WAT Usage

Participants’ basic demographic information, including sex, age, country of residence, and education level, was collected through 4 items. They were asked about their current or former use of a WAT, leading to respective survey variations. Questions also covered the make and model of their WAT (open-ended), duration of usage, and, for current users, the intended continuation period.

#### Perceptions of Features

A total of 28 items asked current users about the frequency and perceived usefulness of common WAT features like active minutes, steps, stairs, sleep, heart rate, energy burned, energy consumed (food), meditation, workouts, calls or texts, contactless payment, calendar reminders, device connectivity, and data sharing. Responses were recorded on a 5-point Likert scale.

#### Behavior Change

Participants’ perceptions of health behavior change due to WAT use were explored through 4 items. A total of 3 items on a 5-point Likert scale measured agreement on improvements in eating habits, physical activity, and sleep. In addition, 1 item presented 4 graphical physical activity patterns for participants to identify their own physical activity changes while using a WAT.

#### Motivation for Wearing a WAT

Participants chose their main motivation for using a WAT from 8 options, including fitness improvement, health monitoring, appearance, activity tracking, social sharing, competition, technology interest, or other (open-ended).

#### Data Usage and Sharing

A total of 2 items used a 5-point Likert scale to explore the usefulness of WAT data for short-term and long-term self-monitoring. Participants were asked what social networks they share their activity data in and could select from 6 options (tick all that apply), including “Facebook,” “Twitter,” “Instagram,” “Within the activity tracker’s software,” “I don’t share my activity data,” or “Other (please specify).” Participants were then asked why they share their data and could choose from 6 options (tick all that apply), including “To share progress,” “To compete with friends,” “To get encouragement from others,” “To motivate others,” “I didn’t share my data,” or “Other (please specify).”

#### Practical Issues

A total of 3 questions explored practical issues related to using WATs. Both current and former users were asked if they had any complaints about their WAT and were asked to select all that applied from a multiple choice question, with responses including “Technical issues,” “It falls off,” “It doesn’t fit,” “It is uncomfortable,” “Lost it,” “Low battery life,” “General wear and tear,” “It often doesn’t match my outfit,” “Problems with the screen,” “Problems uploading the data to supporting software,” “Problems interpreting the data,” “Problems with navigation of supporting website and technology,” “Inaccurate at recording data,” and “Other (please specify).” Participants were asked to rate the extent to which they agreed their experience using a WAT had been positive. Finally, former users were asked to specify their reasons for discontinuing WAT use from a list including device malfunction, insufficient use, technical difficulties, unmet goals, personal dislike, psychological impacts, and others (open-ended).

#### Data Quality Checking

After data collection, signs of internet bot activity were detected in the MTurk responses, notably identical and nonsensical answers to open-ended questions (eg, responding “GOOD” to a question asking what brand and model of WAT they use). To ensure data integrity, we implemented quality-check processes as recommended by Griffin et al [25]. This involved removing responses with a Qualtrics reCAPTCHA score below 0.5, indicating a higher risk of bot activity (on a scale of 0-1, where 1.0 indicates a low risk of bots, and 0.0 indicates a high risk of bots [26,27]). Furthermore, responses completed unrealistically quickly (less than 5 minutes) were excluded, considering the average completion time was 17 minutes. In addition, responses with less than 5% survey completion were removed. These quality-check processes filtered out all the participants initially suspected of being nongenuine.

#### Analysis

Data were downloaded into SPSS (version 26; IBM Corp) for analysis. Categorical variables and ordinal variables were analyzed using the frequency of responses and percentages, while continuous variables were analyzed using medians, means, and ranges. Comparison between 2023 and 2016 WAT users were examined using chi-square tests, with an alpha of .05 used to denote statistical significance.

### Ethical Considerations

Ethical approval for this cross-sectional, descriptive online survey study was provided by the University of South Australia Human Research Ethics Committee (204908). Data collection took place from February to May 2023. As per ethical requirements, the cover page of the survey contained detailed information about the study, and completion of the online survey was regarded as informed consent. Survey data were de-identified.

## Results

### Demographic Characteristics and WAT Usage

A total of 827 survey responses were received. However, 338 were excluded with suspected bot activity (reCAPTCHA scores below 0.5 [n=208] and responses completed in less than 5 minutes [n=130]), and a further 14 responses were excluded for having less than 5% of the survey completed. Consequently, the analytic sample comprised 475 participants (280 recruited through MTurk and 195 recruited through Facebook), with 387 (82%) current users and 88 (18%) former users, which is similar to the 2016 survey proportions of 84% (200/237) current and 16% (37/237) former users among 237 participants.

Table 1 presents the participants’ characteristics. The majority (219/475, 46.1%) were aged 25-34 years, and 60.8% (289/475) were male. This contrasts with the 2016 survey, where the majority were aged 18-24 years (75/237, 33.8%), and a greater proportion (168/237, 70.9%) were female. The 2023 survey was international, with 62% (292/475) of respondents from the United States, 26% (122/475) from Australia, and 12% (61/475) from other countries including Albania, Armenia, and India. In contrast, the 2016 survey was national, with all participants from Australia.

**Table 1:** Demographic characteristics of participants.

Demographic characteristics			2023 survey (n=475), n (%)		2016 survey (n=237), n (%)		*P* value			Chi-square (*df*)	
**Gender**								<.001			64.6 (2)
	Male	289 (60.8)		69 (29.1)							
	Female	185 (38.9)		168 (70.9)							
	Nonbinary or third gender	1 (0.2)		—^a^							
**Age (years)**								<.001			47.8 (5)
	18-24	69 (14.5)		75 (33.8)							
	25-34	219 (46.1)		56 (25.2)							
	35-44	110 (23.2)		47 (21.2)							
	45-54	57 (12)		28 (12.6)							
	55-64	15 (3.2)		14 (6.3)							
	≥65	5 (1)		2 (1)							
**Country**								<.001			349.3 (2)
	United States of America	292 (61.5)		—							
	Australia	122 (25.7)		237 (100)							
	Other	61 (12.8)		—							
**Education**								<.01			13.2 (4)
	Primary school	2 (0.4)		—							
	High school	119 (25.1)		52 (21.9)							
	Vocational qualification, trade school, community college, certificate, diploma or apprenticeship	49 (10.3)		42 (17.7)							
	University bachelor’s degree	232 (48.8)		95 (40.1)							
	Postgraduate degree (eg, Master’s degree)	73 (15.4)		48 (20.3)							

^a^Not available.

The 2023 survey results showed that Apple (210/475, 44%) was the most popular WAT brand, followed by Fitbit (101/475, 21%), Garmin (92/475, 19%), Samsung (47/475, 10%), and other brands (22/475, 5%). This was a significant change from 2016, where Fitbit dominated (160/237, 68%), followed by Garmin (39/237, 17%), Apple (8/237, 3%), Samsung (4/237, 2%), and other brands (26/237, 11%; *P*<.0001). The primary method of acquisition in both years was purchase by the users themselves (2023: 232/475, 49%; 2016: 134/237, 57%) or as gifts from family members (2023: 136/475, 29%; 2016: 103/237, 44%).

In terms of usage duration, current 2023 users reported using their WATs for a median of 18 months, while former users had used them for a median of 9 months. In 2016, current users had a median usage duration of 7 months, and former users had used theirs for 5 months.

When current users from the 2023 survey were asked how long they planned to use their WAT in the future, the most common response was “indefinitely” (82/475, 21%). Similarly, current users of the 2016 survey were also asked how long they planned to use their WATs; the most common response was >36 months, which was the maximum duration response option in that survey. Among former 2023 users, when asked if they would be inclined to ever use a WAT again, 45% (39/88) somewhat agreed, and 40% (34/88) were neutral.

### Motivation and Behavior Change

In 2023, the most commonly cited motivations for using WATs were improving health (239/475, 50%), monitoring activities (221/475, 47%), and enhancing fitness (180/475, 38%; Table 2). The same 3 motivations were common in the 2016 survey, though in a different order, with 2016 participants prioritizing monitoring activity patterns (85/237, 36%), improving fitness (65/237, 27%), and improving health (43/237, 18%). Nearly one-third of 2023 respondents (147/475, 31%) reported improving their appearance as a motivation for using a WAT, compared with <1% in 2016. During analysis of the motivation data, we noticed that the 2023 survey allowed multiple responses for motivation, differing from the 2016 survey’s single-choice format. Given this discrepancy, direct comparisons using chi-square tests were not undertaken.

**Table 2:** Participant motivations for wearing a wearable activity tracker.

Primary motivation	2023 survey (N=475), n (%)	2016 survey (N=237), n (%)
To improve health	239 (50.3)	43 (18.1)
To monitor activities	221 (46.5)	85 (35.9)
To improve fitness	180 (37.9)	65 (27.4)
To improve appearance	147 (30.9)	2 (0.8)
To share my activity	126 (26.5)	—^a^
To compete with family or friends	60 (12.6)	7 (2.9)
To keep up with new technology	45 (9.5)	4 (1.7)
Other	15 (3.9)	15 (6.3)

^a^Not available.

Table 3 presents the comparison of participants’ ratings (percentage of participants responding somewhat agree or agree strongly) for the usefulness of various WAT metrics in 2023 versus 2016. Notably, the perceived usefulness of the step count feature significantly decreased from 94.5% (189/200) in 2016 to 65.9% (255/387) in 2023. Similar declines were seen in the perceived usefulness of activity minutes (151/200, 75.5% in 2016 to 252/387, 65.2% in 2023), and stairs climbed (115/200, 57.5% in 2016 to 189/387, 48.9% in 2023). Conversely, the usefulness rating for energy intake rose from 36% (72/200) in 2016 to 49.6% (192/387) in 2023. The heart rate feature also showed a nonsignificant trend for increased perceived usefulness, from 63% (126/200) in 2016 to 70.5% (273/387) in 2023. In fact, it was the top-rated useful feature in 2023. Ratings for sleep features fell slightly (232/387, 59.9% in 2023 vs 132/200, 66% in 2016) while energy burned remained fairly stable.

**Table 3:** Participant rating of usefulness of features (percentage reporting somewhat agree or agree strongly).

Feature	2023 survey (n=387), n (%)	2016 survey (n=200), n (%)	*P* value	Chi-square (*df*)
Heart rate	273 (70.5)	126 (63)	.06	3.4 (1)
Steps	255 (65.9)	189 (94.5)	<.001	58.5 (1)
Active minutes	252 (65.2)	151 (75.5)	.01	6.6 (1)
Sleep	232 (59.9)	132 (66)	<.001	16.6 (1)
Energy burned	227 (58.6)	113 (56.5)	.61	0.09 (1)
Energy consumed	192 (49.6)	72 (36)	<.001	9.8 (1)
Stairs	189 (48.9)	115 (57.5)	.04	3.9 (1)

Current WAT users in 2023 generally agreed on the usefulness of updated WAT features, with high ratings for workouts (290/387, 75% somewhat or strongly agree), device connectivity (263/387, 68%), receiving calls or text messages (249/387, 64%), contactless payment (200/387, 52%), calendar reminders (214/387, 55%), data sharing (193/387, 50%), and meditation (193/387, 50%).

Respondents were asked their opinions on the usefulness of real-time monitoring and long-term monitoring features. While the majority of 2023 participants somewhat or strongly agreed that both real-time monitoring and long-term monitoring were useful (57% and 58% agreement, respectively), the agreement ratings have fallen since 2016, when the ratings for real-time and long-term monitoring were 84% and 72% agreement, respectively.

Participants were asked whether they had changed their lifestyle behaviors as a result of using a WAT (Table 4). In both the 2023 and 2016 surveys, physical activity was the behavior respondents were most likely to report having changed due to their WAT. However, there was a slight reduction over time in the perceived impact of WATs on enhancing physical activity, from 77% (182/237) agreement in 2016 to 63% (300/475) agreement in 2023. In contrast, the perceived positive impact on diet increased from 36% (85/237) in 2016 to 51% (244/475) in 2023. Similarly, the percentage of participants who felt their sleep improved due to using a WAT rose from 22% (52/237) in 2016 to 42% (200/475) in 2023.

**Table 4:** Perceived change in lifestyle behaviors since using a wearable activity tracker (percentage reporting somewhat agree or agree strongly).

Activity	2023 survey (n=475), n (%)	2016 survey (n=237), n (%)	*P* value	Chi-square (*df*)
Physical activity	300 (63)	182 (77)	<.001	13.4 (1)
Diet	244 (51)	85 (36)	<.001	15.2 (1)
Sleep	200 (42)	52 (22)	<.001	28.1 (1)

### Social Features and Data Sharing

There has been a marked increase in the use of social media platforms for sharing WAT data (Table 5). In 2023, Instagram was used most by 44% (171/387) of participants, a sharp rise from just 1% (2/200) in 2016. Facebook and Twitter saw similar surges, with 36% (141/387) using Facebook (up from 7/200, 4% in 2016) and 35% (134/387) using Twitter (up from 1/200, 1% in 2016). All these increases were statistically significant. Conversely, the proportion of participants not sharing their activity data dropped significantly from 65% (130/200) in 2016 to 27% (104/387) in 2023.

The motivations behind sharing data have also evolved. In 2023, common reasons included sharing progress (145/387, 38%, up from 18/200, 9% in 2016), receiving encouragement (137/387, 35%, up from 13/200, 7% in 2016), and motivating others (138/387, 36%, up from 17/200, 8.5% in 2016). Competing with friends was noted by 35% (134/387) in 2023, a significant increase from 17% (33/200) in 2016.

**Table 5:** Sharing platforms and reasons for sharing data^a^.

		2023 survey (n=387), n (%)			2016 survey (n=200), n (%)			*P* value			Chi-square (*df*)
**Sharing platform**											
	Instagram		171 (44.1)			2 (1)			<.001		118.3 (1)
	Facebook		141 (36.4)			7 (3.5)			<.001		75.8 (1)
	Twitter		134 (34.6)			1 (0.5)			<.001		125.8 (1)
	I don’t share my activity data		104 (26.8)			130 (65)			<.001		79.9 (1)
	Social network within the wearable’s software		113 (29.2)			70 (35)			.15		2 (1)
	Strava		5 (1.3)			9 (4.5)			.01		5.8 (1)
	Other		3 (0.8)			3 (1.5)			.40		0.6 (1)
**Reason for sharing data**											
	To share progress			145 (37.5)			18 (9)			<.001	53.2 (1)
	To get encouragement from others			137 (35.4)			13 (6.5)			<.001	57.8 (1)
	To motivate others			138 (35.6)			17 (8.5)			<.001	50 (1)
	To compete with friends			134 (34.6)			33 (16.5)			<.001	21.2 (1)
	I don’t share data			104 (26.9)			154 (77)			<.001	78.6 (1)
	Other (please specify)			7 (1.8)			4 (2)			.87	0.2 (1)

^a^Participants were allowed to select multiple responses, and percentages reflect the number of participants who selected each response option as a portion of all participants in that survey sample.

### Technical Issues and Barriers

The majority of 2023 respondents agreed that they had an overall positive experience using their WAT (322/475, 68% somewhat agree or agree strongly), though this was a decrease compared with 2016 when 212 of 237 (89%) reported an overall positive experience (*χ^2^_1_*= 36.2; *P*<.001). The most common complaints in 2023 were short battery life (reported by 201/475, 21% of participants), technical issues (cited by 91/475, 19%), and general wear and tear (mentioned by 86/475, 18%; Table 6). Generally, 2023 participants appeared to report slightly more complaints than 2016 respondents. In contrast to this trend, problems with data uploading and inaccurate data recording have decreased slightly over time, from 19% (45/237) in 2016 to 10% (47/475) in 2023, and from 17% (39/237) in 2016 to 7% (32/475) in 2023, respectively.

**Table 6:** Complaints about wearable activity tracker (WAT)^a^.

Complaint	2023 survey (n=475), n (%)	2016 survey (n=237), n (%)	*P* value	Chi-square (*df*)
None	162 (34.1)	71 (30)	.26	1.2 (1)
Short battery life	102 (21.4)	48 (20)	.7	0.1 (1)
Technical issues	91 (19.1)	29 (12.2)	.02	5.4 (1)
General wear and tear	86 (18.1)	32 (13.5)	.11	2.4 (1)
It was or is uncomfortable	77 (16.2)	20 (8.4)	<.01	8.1 (1)
It fell or falls off	75 (15.7)	17 (7.2)	<.001	10.4 (1)
It didn’t or doesn’t fit	71 (14.9)	—^b^	<.001	39.3 (1)
It often didn’t or doesn’t match my outfit	64 (13.5)	44 (18.6)	.07	3.1 (1)
Problems with the screen	58 (12.2)	10 (4.2)	<.001	11.6 (1)
Lost it	47 (9.9)	5 (2.1)	<.001	14.1 (1)
Problems uploading the data to supporting software	47 (9.9)	45 (19)	<.001	11.6 (1)
Problems interpreting the data	41 (8.6)	10 (4.2)	.03	4.6 (1)
Problems with navigation of supporting website and technology	35 (7.4)	16 (6.8)	.76	0.9 (1)
Inaccurate at recording data	32 (6.7)	39 (16.5)	<.001	16.6 (1)
Other (please specify)	21 (4.4)	27 (11.4)	<.001	12.2 (1)

^a^Participants were allowed to select multiple responses, and percentages reflect the number of participants who selected each response option as a portion of all participants in that survey sample.

^b^Not available.

Former users were asked to identify why they no longer used their WAT (Table 7). Among former WAT users in the 2023 survey, the main reasons for ceasing use were that their WAT was broken (29/88, 33%), they did not like it (22/88, 25%), or that it was lost (15/88, 17%), difficult to understand (15/88, 17%), or technical difficulties (15/88, 17%). This was different from the 2016 survey, where the main reasons former users ceased using their WATs were that they felt they had learned everything they could from their WAT (11/37, 30%), their WAT was broken (8/37, 22%), or their WAT was not helping them achieve their goals (5/37, 14%). The findings appear to indicate a shift in why users discontinue WAT usage, with increasing reports of disliking the device (up from 2/37, 5% in 2016 to 22/88, 25% in 2023) and understanding difficulties (up to 2/37, 5% in 2016 to 15/88, 17% in 2023), and fewer users stopping because they felt they had learned all they could from the device (down from 11/37, 30% in 2016 to 5/88, 6% in 2023).

**Table 7:** Former user’s reasons for ceasing use of wearable activity tracker (WAT)^a^.

Reasons to stop wearing tracker	2023 survey (n=88), n (%)	2016 survey (n=37), n (%)	*P* value	Chi-square (*df*)
It broke	29 (33.3)	8 (21.6)	.20	1.6 (1)
I didn’t like it	22 (25.3)	2 (5.4)	.01	6.4 (1)
Got lost	15 (17.2)	4 (10.8)	.37	0.7 (1)
It was difficult to understand	15 (17.2)	2 (5.4)	.08	3 (1)
Technical difficulties	15 (17.2)	4 (10.8)	.37	0.7 (1)
I wasn’t using it enough	14 (16.1)	—^b^	.01	6.6 (1)
It wasn’t helping with my goals	12 (13.8)	5 (13.5)	.98	0.0 (1)
I found it intrusive	10 (11.5)	3 (8.1)	.58	0.29 (1)
I was experiencing negative psychological impacts	8 (9.2)	3 (8.1)	.85	0.03 (1)
I learned everything I could	5 (5.7)	11 (29.7)	<.001	13.4 (1)
Other	6 (6.9)	16 (43.2)	<.001	23.8 (1)

^a^Participants were allowed to select multiple responses, and percentages reflect the number of participants who selected each response option as a portion of all participants in that subgroup.

^b^Not available.

## Discussion

### Principal Findings

This study set out to explore changes in user experiences, preferences, and perceived impacts of WATs from 2016 to 2023. We found significant shifts in brand preferences, with Apple overtaking Fitbit as the most popular WAT brand by 2023. The study found that, in general, WATs are now being used for a longer period compared with the previous survey. The sharing of WAT data on social media platforms has surged, indicating a greater integration of these devices into social connectivity and community engagement. While there was a decrease in the perceived effectiveness of WATs in enhancing physical activity, users reported increased positive impacts on diet and sleep management. However, this was tempered by increased complaints about technical issues and discomfort, as well as a decline in overall positive user experience with WATs. In addition, we observed a shift in reasons for discontinuing WAT use, with more users in 2023 ceasing usage due to dissatisfaction with the device or its complexity. Possible reasons for the key differences between responses in 2016 and responses in 2023 (*P*<.001) are summarized in Multimedia Appendix 2 and further discussed below.

The majority of current users in the 2023 survey reported that they had worn their WAT for a median duration of 18 months and were planning on using their WATs indefinitely. This was significantly greater than that of users in 2016, who reported that they had worn their WATs for 7 months and were planning on using them for >3 years. This is likely due to WATs having existed for a longer period, with more metrics and features available, potentially increasing appeal and, therefore, long-term usage. Most former users in 2023 wore their WATs for a median duration of 9 months, with just over one-third reporting that their device breaking was their main reason for ceasing use. These findings confirm evidence that suggests WATs are often abandoned due to technical malfunctions, including broken WATs or battery issues [28]. In contrast, former users of the 2016 survey reported that they had worn their WAT for a median duration of 5 months, with the main reason for ceasing use being that they had learned everything they could. It is possible that enhancements in WATs over time, such as improved health monitoring capabilities and integration with smartphones, have increased the perceived value of the devices and made them more valuable in people’s lives [18]. These findings suggest that continuous innovation in WAT features may play a crucial role in sustaining user interest and reducing disinterest or apathy over time.

Despite the increase in duration of use, fewer users in 2023 reported having a positive experience with their WAT than in 2016, and in general, reported a greater number of complaints. Short battery life was the most reported complaint in both 2016 and 2023, which remains consistent with research that suggests battery capacity is one of the main user complaints and reasons for abandonment [29]. In addition, complaints such as technical issues, wear and tear, comfort, the WAT falling off, problems with the screen, and the ability to interpret the data were more commonly reported in 2023. This increase in complaints seems counterintuitive, given that WATs are now more advanced with more features. Rising expectations may be behind this–as WATs have become more advanced, user expectations might have risen correspondingly, leading users to be more critical of their devices. An alternative explanation is that the inclusion of more features can make WATs more complex to use. This complexity might lead to frustrations, especially among users who prefer simpler, more intuitive interfaces. More advanced devices that offer enhanced features may also explain a shift in user preferences for Apple WATs, with features like call and text, smart pay, and safety functions easily integrated into users’ Apple technology ecosystems.

Whilst WATs track various health behaviors like physical activity, diet, and sleep, physical activity remained the most monitored behavior in both the 2016 and 2023 surveys. It was also the behavior most frequently reported as improved due to WAT use, consistent with research highlighting WAT’s effectiveness in increasing physical activity across different populations [14]. In 2023, there was an increase in perceived change in diet and sleep since using a WAT, suggesting that WATs have the potential to influence a range of lifestyle behaviors, and users may benefit more from the advanced features included in more recent models.

Interestingly, although the perceived change in diet and sleep since using a WAT increased between 2016 and 2023, and the perceived usefulness of energy consumed also increased, sleep was considered less useful in 2023. Heart rate monitoring was considered the most useful feature in 2023, a shift from 2016 when step count was rated highest. These findings may reflect the increased popularity of fitness activities emphasizing heart rate, such as high-intensity interval training, or other activities that do not record steps, such as strength training. Previous research suggests that resting and exercise-related heart rate measures are considered useful as they provide noninvasive and time-efficient insights into aerobic fitness, recovery, and performance [30]. This trend in the evolving usage of WATs, with a focus on heart rate monitoring over steps, suggests a broader shift in user health priorities and fitness trends, mirroring the advancements in wearable technology and its increased capability to provide detailed, fitness-specific metrics that cater to modern exercise regimens and health awareness.

Participants in the 2023 survey were notably more inclined to share data on mainstream social networks like Instagram, Facebook, and Twitter, a change from the 2016 survey. This shift contrasts with earlier research suggesting a preference for sharing on niche physical activity social networks [31,32]. This increase in sharing on broader platforms may be attributed, at least in part, to a continued increase in social media usage from 2.73 billion users in 2017 to 4.59 billion users in 2023 [33]. In particular, Instagram has a strong fitness subculture [34]. The rise of fitness influencers and communities on these platforms may also encourage users to share their data more openly. In addition, there seems to be an evolving perception of sharing fitness data, now viewed as a motivational and supportive tool, possibly amplified by the COVID-19 pandemic’s push for social connectedness during periods of isolation [35].

### Strengths and Limitations

This study has several strengths. First, the longitudinal, cross-sectional design allowed us to compare WAT user experience from 2016 to 2023, offering a valuable perspective on the evolution of user preferences and practices over time. The 2023 study attracted a large and diverse sample, enhancing the generalizability of our findings. We considered the perspectives of both current and former WAT users and examined various facets of WAT usage, including brand preference, feature utility, motivations, social sharing behaviors, and technical issues. In addition, our study was rigorous in its methodology, using an existing survey tool, conducting test-retest analysis, and implementing extensive data quality checks.

Limitations must also be acknowledged. As a survey, data are self-reported, which introduces the possibility of biases as participants may over or underestimate their usage or satisfaction with their WATs due to factors such as factors such as misremembering past experience, social desirability, or acquiescence. Nonresponse bias could also be present. Response options for the item “How long did you use your wearable?” differed between the 2016 and 2023 surveys, which may have introduced some bias. Although the sample was large and diverse, the findings may not be entirely generalizable to all WAT users, especially those in different geographical locations or demographic groups not adequately represented in the sample. There was a very low representation of older adults in both the 2016 and 2023 surveys. This differs from other studies, where older adults are among the most studied groups for acceptability studies. This discrepancy may be explained by our recruitment methods, which utilized social media and MTurk to recruit existing users of WATs. These platforms potentially reach younger and middle-aged adults more readily than older adults. In contrast, many evaluations of mHealth acceptability in older adults are linked to interventional studies where devices are provided directly to participants by researchers. This approach might better engage older adults, who may be less likely to participate in online surveys but more receptive to direct intervention studies. In addition, there were some demographic differences between the survey years that could have impacted the findings. For example, the 2023 survey involved an international sample, whereas the 2016 survey involved only Australians. The 2023 survey also had proportionally more males than the 2016 survey. The use of multivariable analysis methods would have enabled examination of the effects of these cohort differences but was not possible due to the unavailability of the individual-level data from the 2016 survey. We conducted our analyses using the summary data available, which still offers valuable insights into the trends and changes over time. Another limitation of the study includes the presence of numerous internet bots from MTurk that had completed the 2023 survey. Although we conducted data quality checking, concerns have been raised about MTurk, including self-misrepresentation, self-selection bias, English language fluency, and inattention, which may impact a study’s validity [36].

### Implications for Research and Practice

A key overall finding in this study is that the use of WATs is shifting—including a shift in brand preference toward Apple, a shift in the metrics valued by users away from simple physical activity monitoring features, and toward more detailed metrics such as heart rate tracking, diet, and sleep tracking. People are also sharing their data differently, with increased sharing of WAT data on social media platforms like Instagram. These findings could inform behavioral health interventions. Health promotion researchers might consider integrating WATs into health promotion and disease prevention strategies, given their potential to positively influence lifestyle behaviors. There is also the potential for including sedentary behavior as a metric for users to track, given its importance for health and inclusion in physical activity guidelines. In addition, if patients are willing to discuss their WAT information with health professionals, there is the potential for integrating WATs into health care, providing insights into how different activities and behaviors affect health and well-being and facilitating shared goal setting and monitoring. However, researchers and health professionals seeking to use WATs with research participants and patients should be cognizant of the common usability complaints, such as battery life and ease of use, since these aspects may be crucial in the long-term adherence to WAT-based programs.

Future research should look to examine WAT user preferences and practices with diverse populations, including different age groups, geographical locations, and health statuses, to provide a more comprehensive understanding of WAT usage patterns and preferences across a broader demographic. In addition, given the recent increase in sharing WAT data on social media between 2016 and 2023, studies investigating the psychological and behavioral impacts of sharing WAT data on social media platforms would be valuable. Research could explore how social sharing influences motivation, behavior change, and community building in the context of health and fitness. Such findings could influence the development of interventions incorporating the use of WATs for healthy lifestyle behaviors. In addition, given the increase in complaints about technical issues and device comfort, research focusing on user interface design and ergonomic improvements in WATs could be beneficial. This would need to be done in partnership with WAT manufacturers to inform future product development.

### Implications for WAT Design

Our study highlights several key insights that are highly relevant for companies involved in the design and development of WATs. The most common complaint from users at both time points was short battery life. Manufacturers should prioritize advancements in battery technology to extend the life of WATs between charges to enhance user satisfaction and device adherence. In addition, more users in 2023 reported discomfort and issues with the fit of their devices than in 2016. This could be due to increased battery size or change in device shape due to, for example, the addition of heart rate monitors in more models. Companies should focus on ergonomic designs that offer better comfort for long-term wear, such as adjustable bands, lightweight materials, and options for different wrist sizes.

Technical reliability also emerged as a crucial area for improvement. Although less common in 2023 than in 2016, users frequently mentioned issues with data accuracy and connectivity problems. Improving the reliability and accuracy of WAT sensors and ensuring seamless connectivity with other devices are essential for maintaining user trust and encouraging continued usage. Furthermore, the shift in user preference toward advanced health metrics like heart rate monitoring over basic step counts indicates that users are seeking more sophisticated health insights. Companies should continue to develop and highlight features that provide deeper health analytics, such as heart rate variability, stress levels, and sleep quality.

To address these needs effectively, manufacturers should invest in user education and support. Providing comprehensive tutorials, user guides, and customer support can help mitigate issues related to device complexity and usability, enabling users to understand and make the most of their WAT features. By addressing key areas such as battery life, comfort, technical reliability, and advanced health metrics, companies can enhance the user experience and ensure long-term device adoption, positioning WATs as valuable tools for health and fitness.

### Conclusions

In conclusion, this study has provided insights into the evolving landscape of WAT usage from 2016 to 2023. The shift in brand preference toward Apple and the longer usage duration indicate a maturing market and evolving user needs. Notably, the surge in sharing WAT data on platforms like Instagram highlights the increasing social aspect of health monitoring. However, despite technological advancements, the rise in user complaints and the shift in reasons for discontinuation emphasize the need for improvements in WAT design and functionality. These trends underscore the growing importance of integrating sophisticated health metrics such as heart rate monitoring, which aligns with the rising popularity of specific fitness activities and a broader shift in health and wellness culture. As WATs become more embedded in users' daily lives, their role in health promotion and behavioral change becomes increasingly significant. Future research should continue to explore these dynamics, particularly focusing on diverse populations and the psychological impacts of social sharing, to further understand and optimize the potential of WATs in supporting users’ health and well-being.

## Appendix

Multimedia Appendix 1 Survey instrument.

Multimedia Appendix 2 Summary of key changes between 2016 and 2023 and related insights.

## Abbreviations

MTurk: Mechanical Turk
WAT: wearable activity tracker

## Data Availability

The datasets used and/or analyzed during this study are available from the corresponding author on reasonable request.
