# Supplementary figures and images for: Changing User Experience of Wearable Activity Monitors Over 7 Years: Repeat Cross-Sectional Survey Study
Source: J Med Internet Res. 2025 Feb 13;27:e56251. doi: 10.2196/56251 (PMC11888053; doi:10.2196/56251)

**
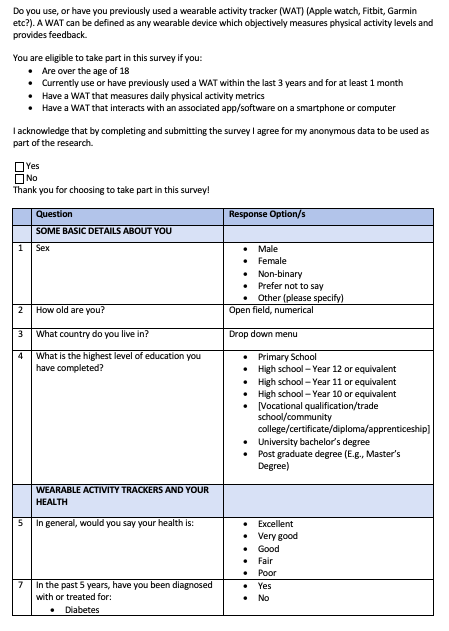

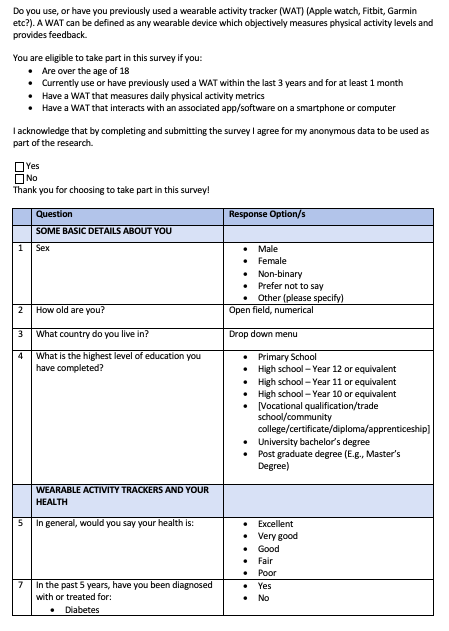
Supplementary file: Survey Instrument**

**
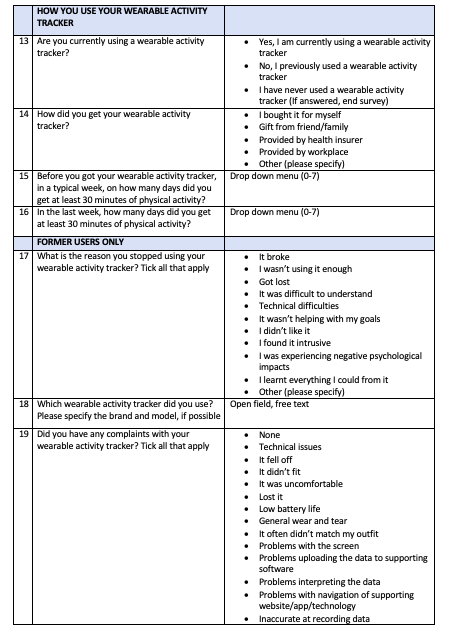
**

**
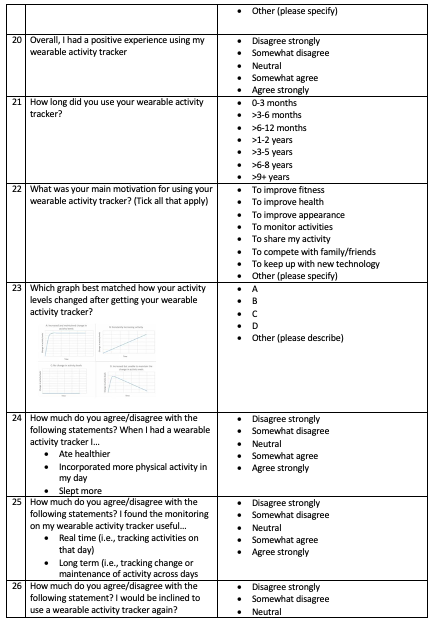
**

**
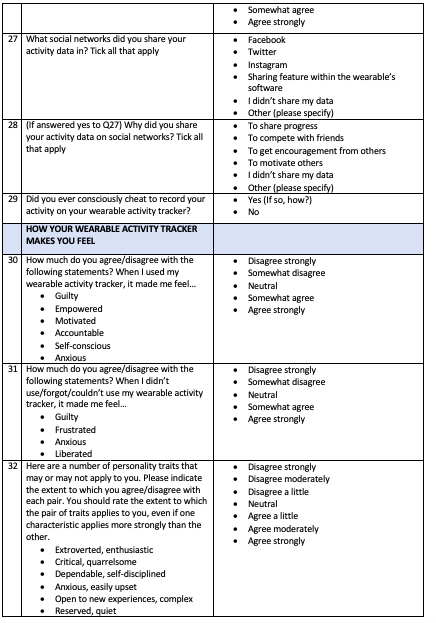
**


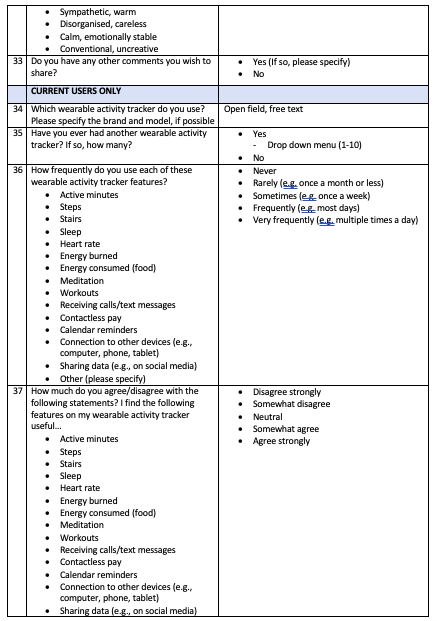


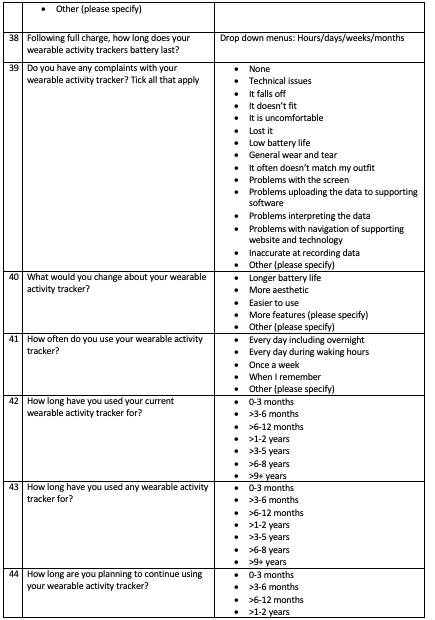


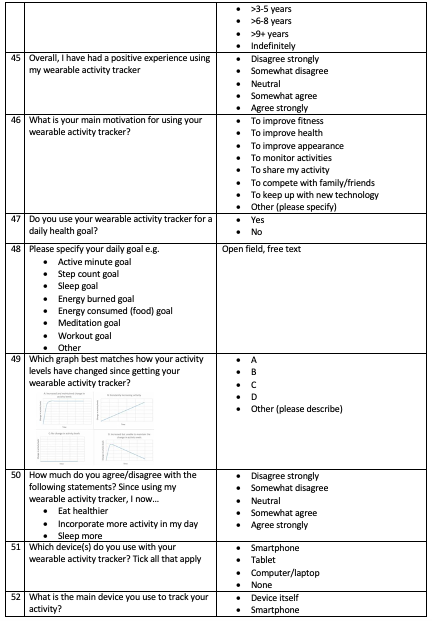


**
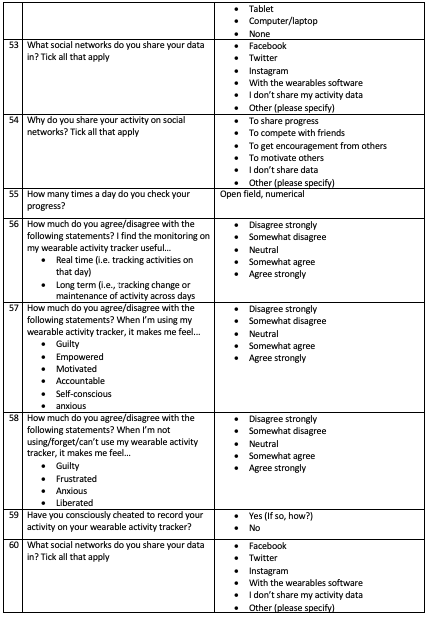
**


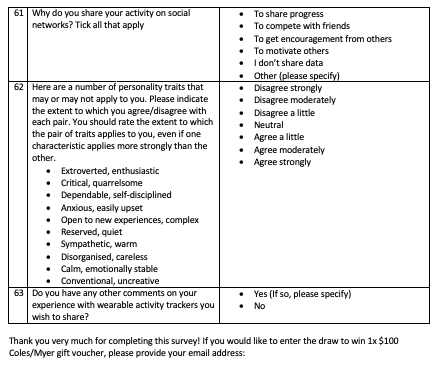


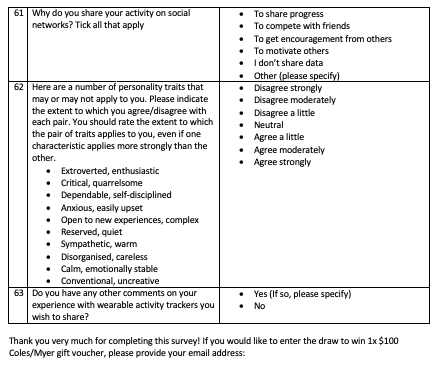

Supplement: Multimedia Appendix 1 [file jmir_v27i1e56251_app1.docx]
