# Supplementary material for: Changing User Experience of Wearable Activity Monitors Over 7 Years: Repeat Cross-Sectional Survey Study
Source: J Med Internet Res. 2025 Feb 13;27:e56251. doi: 10.2196/56251 (PMC11888053; doi:10.2196/56251)
Supplement: Multimedia Appendix 2 [file jmir_v27i1e56251_app2.docx]

**Supplementary file 2:** Summary of key changes between 2016 and 2023 and related insights

| **Category** | **Item** | **2023 result compared to 2016** | **Category insight** |
| --- | --- | --- | --- |
| Usefulness of features | Steps | Less useful in 2023 | The finding that steps were considered less useful in 2023 may reflect the increased popularity of alternative fitness activities such as strength training or HIIT training, which are better recorded using other metrics such as heart rate.  Sleep tracking has potentially become less novel in 2023, whereas there is increased integration of third-party diet apps with WAT apps.  WAT manufacturers should continue to develop features that provide deeper health analytics and integration with other health apps. |
|  | Sleep | Less useful in 2023 |  |
|  | Energy consumed | More useful in 2023 |  |
| Perceived change in lifestyle behaviours since using a WAT | Physical activity | Less likely in 2023 | In both 2016 and 2023, perceived changes in physical activity were most likely, followed by diet and then sleep. However, physical activity was less likely to change in 2023 compared to 2016, and diet and sleep were more likely to change in 2023.  WATs have the potential to influence a range of lifestyle behaviours and users may benefit from the additional features and connectivity included in more recent models. |
|  | Diet | More likely in 2023 |  |
|  | Sleep | More likely in 2023 |  |
| Social media sharing | Instagram | More likely in 2023 | The finding that participants were more likely to share their WAT data on social media, particularly on Instagram, Facebook, and Twitter, in 2023 may broadly reflect increased social media use.  Instagram showed the biggest increase. Instagram is a popular platform for fitness influencers and content, which may encourage users to share their own data.  Research should examine the impacts (behavioural and psychological) of sharing WAT data on social media platforms. Understanding these effects (whether positive or negative) may have implications for interventions using WATs. |
|  | Facebook | More likely in 2023 |  |
|  | Twitter | More likely in 2023 |  |
|  | Does not share | Less likely in 2023 |  |
| Complaints | It fell/falls off | More likely in 2023 | While short battery life was the top complaint in both years, there were some differences between 2016 and 2023.  There were fewer complaints about data accuracy and device connectivity, suggesting technical advancements have been made, but issues remain. Additionally, there was an increase in complaints about device fit. This may be due to larger batteries needed to power more complex devices or a difference in shape due to the addition of features such as heart rate monitors on more models. More users reported that their device fell off and that they lost the device.  Manufacturers should prioritise advancements in battery technology and ergonomic designs that offer better comfort for long-term wear. Continued improvements in reliability and accuracy are needed. |
|  | It didn't/doesn’t fit | More likely in 2023 |  |
|  | Problems with the screen | More likely in 2023 |  |
|  | Lost it | More likely in 2023 |  |
|  | Problems uploading data | Less likely in 2023 |  |
|  | Inaccurate | Less likely in 2023 |  |
| Positive experience | Overall positive experience | Less likely in 2023 | The finding that users were less likely to have an overall positive experience with their WAT in 2023 could be caused by a range of factors, e.g.,   - increased issues with WAT fit and falling off (as discussed above) - increased expectations (i.e. due to perceived technological advances) that were not met. - increased complexity (e.g., due to the addition of new features and more complex accompanying apps)   User experience may be improved by providing clear instructional documentation and increased customer support. |
| Former users’ reason for stopping use | I learnt everything I could | Less likely in 2023 | “I learnt everything I could” went from the most common reason to stop using a WAT (except for “other”) in 2016 to the least common reason to stop using a WAT in 2023.  Early trackers focussed largely on step count. More recent models include additional features such as heart rate monitoring and GPS tracking. Accompanying smartphone apps also include additional content, such as example workouts. This has increased the information available to users and potentially their perceived value and prolonged use. |

*Note:* This table summarizes results with a significant difference between 2016 and 2023 at *p* < .001. WAT = wearable activity tracker.
